# Perceived Stigma and Associated Factors Among Patients with Tuberculosis and Their Families in Jazan Region, Saudi Arabia

**DOI:** 10.3390/healthcare13172120

**Published:** 2025-08-26

**Authors:** Anas Talal Al-Rajhi, Ahmad Y. Alqassim

**Affiliations:** 1 Joint Program for Preventive Medicine (The Saudi Board), Jazan Health Cluster, Jazan 45141, Saudi Arabia; 2 Family Medicine Department, Jazan University Hospital, Jazan 45142, Saudi Arabia; aalqassim@jazanu.edu.sa

**Keywords:** tuberculosis, perceived stigma, risk factors, family members, Saudi Arabia

## Abstract

**Background:** Stigma is a major barrier to tuberculosis (TB) control worldwide. However, there is limited evidence of TB-related stigma not only toward patients but also toward their family members in Saudi Arabia. This study aims to assess the level of TB-related stigma and associated factors among individuals with TB and their families in Jazan, Saudi Arabia. **Methods:** A cross-sectional survey was conducted among 404 participants (272 adult patients with TB and their 132 family members). Participants were interviewed using a structured questionnaire adapted from validated TB-related stigma scales, covering sociodemographic factors and perceived stigma. Sociodemographic factors were used to compare stigma grades. Collected data were analyzed using the Statistical Package for the Social Sciences. Frequencies and percentages were used to describe qualitative variables, while the χ^2^-test was applied to compare TB-related stigma levels according to demographic factors. *p* < 0.05 was considered statistically significant. **Results:** Most participant patients had pulmonary TB (78.7%), while 21.3% had extrapulmonary TB. More than half of them (50.7%) experienced severe stigma, while 23.5% had mild stigma. Levels of TB-related stigma differed significantly according to the patients’ age groups (*p* = 0.011), residence (*p* < 0.001), occupation (*p* = 0.022), and type of TB, which was higher among those with pulmonary TB (*p* = 0.003). Moreover, 24.2% of family members experienced severe stigma, while 25% had mild stigma. Perceived stigma showed a negative impact on the management of TB. Levels of stigma differed significantly among family members according to their residence (*p* < 0.001) and marital status (*p* = 0.018). **Conclusions:** TB-related stigma is widespread among individuals with TB and their family members in Saudi Arabia. This stigma has significant negative impacts on the management of TB. Levels of perceived stigma are higher among younger patients, those living in urban areas, unemployed patients, and patients with pulmonary TB. Among family members, the stigma levels are higher for those living in urban areas and single individuals.

## 1. Introduction

Tuberculosis is a chronic infectious disease caused by *M. tuberculosis* and is a leading cause of morbidity and mortality worldwide [1,2]. During the 20th century, the TB incidence, prevalence, and case fatality rates fell steadily in developed countries. This could be attributed to prevention measures, along with good public health practices and better treatment [3]. However, subsequent efforts reversed this decline, with reports indicating that one patient dies globally every 20 s [4].

In 2023, an estimated 10.8 million people contracted TB worldwide. The global TB incidence rate was 134 per 100,000, with an estimated 1.25 million deaths. Although the net reduction in the global TB incidence rate between 2015 and 2023 was 8.3%, it remains far from the WHO End TB Strategy milestone of a 50% reduction by 2025 [5].

Global efforts to combat TB have saved about 66 million lives since 2000. These efforts include focusing on early case detection, standardized treatment (e.g., Directly Observed Treatment, Short-course), health system strengthening, and addressing MDR-TB and HIV co-infection. However, the COVID-19 pandemic reversed several years of successful efforts to reduce TB and, for the first time in over a decade, TB deaths increased in 2020 [6].

Tuberculosis remains a key public health concern in the Kingdom of Saudi Arabia (KSA), which is a global focal point for the influx and outflux of travelers and expatriates who constitute a large portion of the population. Therefore, the KSA is vulnerable to the international spread of TB but may also become a hotspot, especially for multidrug-resistant TB [7].

Stigma is a socially constructed process that stems from perceived differences, power dynamics, and social exclusion [8]. Social (enacted) stigma refers to experiencing discrimination by other people based on perceived social inferiority, whereas perceived stigma refers to a sense of unworthiness, guilt, and the shame and expectation of discrimination that prevent people from discussing their experiences [9].

Initially, Goffman [10] described “stigma” as a negative or discrediting trait that diminishes an individual’s standing in society. Building upon this, Weiss [11] characterized “health-related stigma” as a socially constructed process or a personal experience marked by exclusion, rejection, blame, or devaluation. Such experiences often stem from actual or anticipated negative societal judgments directed toward individuals or groups associated with specific health conditions. Visser et al. [12] further elaborated on this definition by identifying two fundamental components: the direct experiences of discrimination encountered by individuals within their communities, and the fear or anticipation of such social repercussions, regardless of whether they have occurred. Thus, framing stigma as a social process emphasizes the significance of interpersonal dynamics between individuals affected by a health condition and those who are not.

Thomas and Stephen [13] stated that TB is highly stigmatized, which can be experienced in different social settings such as home, workplace, and community. Perceived stigma has a considerable impact on health, leading patients to refuse medical services by discouraging health-seeking behaviors. This leads to the distortion of health conditions, making the disease difficult to treat and increasing its infectivity and communicability. It is more common in patients with TB.

TB is viewed as a stigmatizing disease because of its associations with several marginalized groups, e.g., the poor [14], ethnic minorities, low social class members [15], prisoners and refugees, and those with HIV/AIDS [16]. Moreover, the impact of stigma can be felt at home, in the workplace, at health facilities, and in the community [17].

Although the treatment of TB is affected by various biological, cultural, and economic factors, TB-related stigma continues to be a major social factor affecting patients’ compliance with treatment and influencing their health-seeking behaviors. Therefore, the proper understanding of TB-related perceived stigma is crucial to improving the quality of life of patients with TB [18]. Nevertheless, studies exploring TB-related stigma toward patients and their family members in the KSA are scarce. Therefore, this study aims to assess the level of TB-related stigma and its associated factors among patients and their families in the Jazan Region, KSA.

## 2. Methods

Following a cross-sectional research design, the sampling frame included all registered patients with TB and their family members in the Jazan Region, Saudi Arabia.

All currently registered active TB cases at the Tuberculosis Control Unit, Jazan Health Sector, Jazan City comprised the study population. However, only adult patients and their family members aged over 18 years, who had been registered with TB for at least one year, were included in the study.

The sample size was calculated according to the following formula: (n = z^2^.p.q/d^2^) [19], where (n) is the minimum sample size of participants; (z) = 1.96; the assumed prevalence (p) of stigma among TB patients was 41.8% according to Eldahshan et al. [20]; and the acceptable margin of error (d) was set at 5%. Based on this formula, the recommended sample size was 374 participants. To account for possible non-responses and missing data, the sample size was increased to 404 participants (272 TB patients and 132 family members), selected using a simple random sampling technique.

A structured questionnaire for patients with TB was developed by the researcher. It was adapted from the scales developed by Van Rie et al. [21] and Arcêncio et al. [22]. The questionnaire had an excellent internal consistency (Cronbach’s alphas 0.82–0.91) and moderate test–retest reliability, and the construct validity showed an inverse correlation with social support (21). The study questionnaire was translated into the local Arabic language by the researcher for easy understanding by participants in the Jazan Region.

The study questionnaire comprised 12 statements. Responses were scored according to a 5-point Likert scale, ranging from 0 = strongly disagree to 4 = strongly agree. The sum of the 12 scores quantified the perceived TB-related stigma, which ranged from 0 to a maximum of 48. Respondents whose TB-related stigma total scores were below 13 were considered to have no stigma. Those with stigma scores ranging from 13 to 24 were considered to have mild stigma, and those with stigma scores above 24 were considered to have severe stigma.

A pilot study was conducted in 20 people to test the data collection tool and assess the time needed to fill out the questionnaire. The results of the pilot study guided the researcher toward the final form of the tool.

Ethical approvals were obtained from the Institutional Review Board (IRB) at the General Directorate of Health in the Jazan Region. Written informed consent was obtained from each participant. Participation remained anonymous, and data were treated with full confidentiality.

Collected data were analyzed using SPSS version 28 (IBM Corp., Armonk, NY, USA) Frequencies and percentages are used to describe qualitative variables. The Chi-square (χ^2^) test was applied to compare TB-related stigma levels according to demographic factors. *p*-values < 0.05 were considered statistically significant.

## 3. Results

Table 1 lists the personal characteristics of participants with TB (n = 272) and their family members (n = 132).

Table 2 shows different aspects of perceived TB-related stigma among patients with TB.

**Figure 1:**
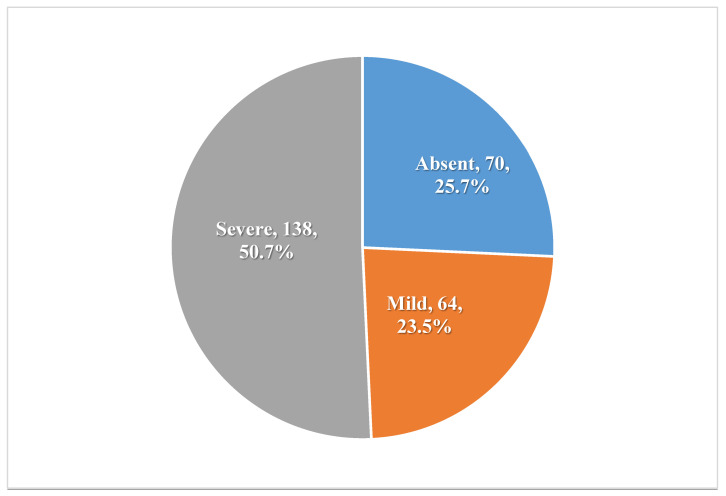
Levels of TB-related stigma among participants (n = 272).

Figure 1 indicates that 50.7% of patients with TB experienced severe stigma, while 23.5% had mild stigma.

Table 3 shows that the main negative impacts of perceived stigma on the management of TB included hindering the utilization of TB services (21.3%) and continuing to receive TB services (22.1%). Additionally, embarrassment at being seen by neighbors prevented patients from seeking and accessing TB services (19.1%), as well as preventing them from seeking and obtaining TB services at home (14%) and at work (18.4%). Moreover, perceived stigma prevented seeking timely care, receiving an accurate diagnosis, starting treatment, committing to treatment, or completing treatment (24.3%). In addition, perceived TB-related stigma was associated with denial of access to services (25.7%) and divorce due to infection with TB (1.5%). Therefore, 36.8% of patients wished to see a change in TB services, laws, and policies to address the sense of shame and embarrassment related to TB.

Table 4 indicates that levels of TB-related stigma differed significantly according to patients’ age groups (*p* = 0.011), being the highest among young patients (aged <25 years). TB-related stigma also differed significantly according to the patient’s residence (*p* < 0.001), being higher among those living in urban areas. TB-related stigma differed significantly according to patients’ occupations (*p* = 0.009), being the highest among patients who were unemployed. Moreover, it differed significantly according to the type of TB, being higher among those with pulmonary TB (*p* = 0.003).

Table 5 shows different aspects of perceived TB-related stigma among family members of patients with TB.

**Figure 2:**
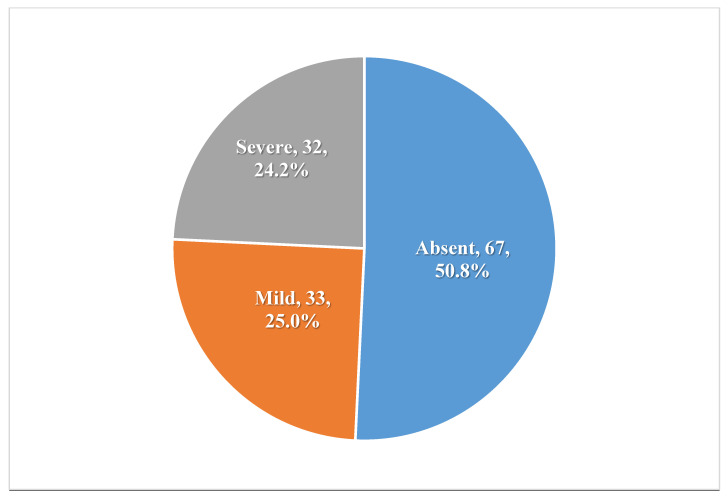
Levels of perceived stigma among family members.

Figure 2 indicates that 24.2% of family members experienced severe stigma, while 25% had mild stigma.

Table 6 shows that levels of perceived TB-related stigma differed significantly among family members according to their residence (*p* < 0.001), being higher among those living in urban areas. TB-related stigma also differed significantly according to their marital status (*p* = 0.018), being highest among single individuals.

Table 7 shows that levels of perceived TB-related stigma differed according to the study group, with participant patients with TB experiencing a significantly higher severe grade than those experienced by participant family members (50.7% and 24.2%, respectively, *p* < 0.001).

## 4. Discussion

The present study revealed that perceived TB-related stigma is very common among patients with TB and their family members in the Jazan Region, Saudi Arabia. Our results showed that more than half of the patients experienced a severe stigma, while about one-fourth faced a mild stigma. On the other hand, about half of their family experienced that stigma (24.2% had severe stigma, while 25% had mild stigma).

This finding is in accordance with those reported in several other studies. Ali et al. [23] reported that more than one-fourth of Indian patients with TB (26.75%) had TB-related stigma. In Egypt, Eldahshan et al. [20] reported that the prevalence of stigma among individuals with TB was 41.5%. Over half of the patients with TB in the urban slums of Uganda (52%) had high stigma [24]. Stanikzai et al. [25] reported a higher prevalence of stigma among patients (52%) in Afghanistan.

Nuttall et al. [26] explained these findings by stating that the prevalence of TB-related stigma varies geographically and by residence. In specific subpopulations, it may affect up to 80% of patients.

Our study showed that TB-related stigma, whether perceived at home or work, had negative impacts on disease management, hindering access to or discontinuing patients’ seeking and receiving TB healthcare services. Moreover, TB-related stigma was associated with family disruption (e.g., divorce). Therefore, the participants hoped to overcome the sense of shame and embarrassment related to TB.

Thomas and Stephen et al. [13] argued that TB is a highly stigmatized disease, which can be experienced in different social settings, e.g., at home, in the workplace, and in the community. Its significant impact on health encourages patients to deny the disease and avoid accessing healthcare services through discouraging health-seeking behavior. This leads to a distortion of their health, making the disease difficult to treat and increasing its infectivity and communicability.

Ashaba et al. [24] added that TB-related stigma is a significant barrier to TB control. Individuals diagnosed with TB frequently express anxiety regarding potential experiences of discrimination, social isolation, and rejection. They may also fear consequences such as marital dissolution, reduced opportunities for marriage, restrictions on sharing food or utensils within the household, and becoming targets of community gossip. These concerns can contribute to delays in seeking medical attention for TB symptoms and may negatively influence adherence to treatment regimens. Consequently, stigma associated with TB is widely recognized as a significant obstacle to effective TB control efforts [27].

Kane et al. [28] stressed that TB-related stigma is influenced by limited knowledge about TB’s cause, transmission, diagnosis, and treatment. They added that health outcomes are hindered by the stigmatization of TB, which affects healthcare and its delivery. TB-related stigma affects social status, damages family reputation, and negatively impacts employment. Hence, there is a high prevalence of unemployment among patients and reduced marital prospects, with a high prevalence of single patients. Moreover, TB-related stigma toward drug-resistant TB has a different effect on the outcome of the disease, being more blamed, shamed, and self-stigmatized due to healthcare workers’ assumption that non-adherence to therapy caused it.

Alema et al. [29] noted that hindered seeking of the necessary healthcare and delayed diagnosis driven by perceived TB-related stigma intensify the infectious pool of TB, increasing the risk of household contact transmission and community transmission.

The present study found that levels of perceived TB-related stigma were significantly more prevalent among younger patients and those living in urban areas and were highest among unemployed TB patients. Moreover, TB-related stigma was significantly higher among those with pulmonary TB. On the other hand, the levels of TB-related stigma perceived by family members were significantly higher among those living in urban areas and single individuals.

Our results are similar to those of other studies. Some of the risk factors linked to perceived TB-related stigma are age, gender, residence, marital status, and monthly income. Higher TB-related stigma among younger patients with TB (aged <25 years) may be explained by unemployment. This also may explain why unemployed individuals experienced higher TB-related stigma. Moreover, patients living in rural areas may experience higher TB-related stigma than those in urban areas, possibly due to a combination of sociocultural, educational, and structural factors [15,23,30].

Eldahshan et al. [20] reported that TB-related stigma differs significantly according to sociodemographic characteristics, being more prevalent among younger patients [43.5%], males [43.9%], married patients [46.7%], and urban residents [57.7%].

De Santo et al. [31] emphasized that a particular concern is the need to avoid community stigmatization of individuals identified with TB. During follow-up visits by survey team members in households of identified TB cases, care must be taken to avoid stigmatizing individuals and household members. The geographical, ethnic, and cultural contexts can heavily influence the potential for stigma. Depending on the risk of being stigmatized, the risk/benefit ratio of the survey project can greatly vary. Survey teams should work with communities to determine the potential for stigma and devise ways to reduce it.

Several studies have reported interventions aiming to reduce perceived TB-related stigma. They stress that psychosocial assistance and educational interventions can be effective in most settings. Moreover, counseling, text messaging, and community campaigns have been applied in some low and middle-income countries [25,32].

The present study determined that the prevalence of perceived severe-grade TB-related stigma among patients was significantly higher than that experienced among family members. This finding may be because the stigma is closely tied to the individual’s identity, visibility, and perceived responsibility for the disease.

Machavariani et al. [33] stated that perceived stigma is a result of the stigmatizing attitudes of other people. Therefore, anti-stigma interventions should be focused not only on individuals within a household, but also in the community and healthcare institutions. Family-based counseling, contact-based health education, awareness-raising campaigns, and policy changes can successfully improve attitudes, reduce stigma, and constitute an effective path to combating TB-related stigma.

To the best of the researchers’ knowledge, this is the first analysis of perceived TB-related stigma among family members of individuals with TB in the KSA. However, a few limitations should be acknowledged. First, the study utilized a cross-sectional design, which is more suitable for hypothesis generation than for hypothesis testing. Second, it was a single-center study conducted in Jazan City, which may limit the external validity and generalizability of the findings. Third, the reliance on self-reported measures to assess TB-related stigma may introduce response bias due to the subjective nature of the responses.

## 5. Conclusions

In conclusion, perceived TB-related stigma is widespread among those with TB and their family members in the Jazan Region, Saudi Arabia. This stigma has a significant negative impact on the management of TB. Higher levels of perceived stigma were observed among younger patients, those living in urban areas, and unemployed individuals. Moreover, the stigma is significantly higher among pulmonary TB patients. On the other hand, the levels of TB-related stigma perceived by family members are higher among those living in urban areas and single individuals. To address this issue, psychosocial support, counseling, text messaging interventions, and community-based educational campaigns are recommended to reduce TB-related stigma.

## Figures and Tables

**Table 1:** Personal characteristics of participants with TB and their family members.

	Patients with TB (n = 272)		Family Members (n = 132)	
Personal Characteristics	No.	%	No.	%
Age groups				
<25 years	61	22.4	34	25.8
25–34 years	88	32.4	44	33.3
35–44 years	77	28.3	34	25.8
45+	46	16.9	20	15.2
Gender				
Male	192	70.6	78	59.1
Female	80	29.4	54	40.9
Residence				
Urban	124	45.6	48	36.4
Rural	148	54.4	84	63.6
Marital status				
Single	144	52.9	68	51.5
Married	110	40.4	58	43.9
Divorced	8	2.9	2	1.5
Widow	10	3.7	4	3.0
Occupation				
Unemployed	138	50.8	70	53.0
Student	24	8.8	12	9.1
Governmental job	54	19.9	20	15.2
Private job	48	17.6	28	21.2
Retired	8	2.9	2	1.5
Family monthly income				
<SAR 5000	172	63.2	74	56.1
SAR 5000–10,000	58	21.3	40	30.3
>SAR 10,000	42	15.4	18	13.6
Type of TB				
Pulmonary	214	78.7	--	--
Extrapulmonary	58	21.3	--	--

**Table 2:** Aspects of perceived TB-related stigma among participant patients.

	Strongly Disagree		Disagree		Neutral		Agree		Strongly Agree	
Aspects of Perceived Stigma	No.	%	No.	%	No.	%	No.	%	No.	%
Feeling annoyed by others’ reaction when they know I have TB	32	11.8	50	18.4	26	9.6	130	47.8	34	12.5
Losing friends when telling them I have TB	36	13.2	50	18.4	32	11.8	128	47.1	26	9.6
Feeling lonely	32	11.8	60	22.1	60	22.1	88	32.4	32	11.8
Keeping distance from others to avoid infecting them	20	7.4	42	15.4	24	8.8	122	44.9	64	23.5
I’m afraid to tell those outside my family that I have TB	36	13.2	52	19.1	18	6.6	124	45.6	42	15.4
I’m afraid to go to TB clinics because others might see me there	74	27.2	86	31.6	24	8.8	76	27.9	12	4.4
I’m afraid to tell others that I have TB because they might think I also have AIDS	50	18.4	82	30.1	52	19.1	50	18.4	38	14.0
I feel guilty that my family bears the burden of caring for me	34	12.5	82	30.1	38	14.0	76	27.9	42	15.4
I choose carefully who to tell about the fact that I have TB	32	11.8	60	22.1	32	11.8	108	39.7	40	14.7
I feel guilty for having TB due to smoking, drinking or other irresponsible behaviors	64	23.5	92	33.8	64	23.5	28	10.3	24	8.8
I’m worried about my potential for AIDS	46	16.9	96	35.3	70	25.7	22	8.1	38	14.0
I’m afraid to tell my family I have TB	54	19.9	106	39.0	24	8.8	74	27.2	14	5.1

**Table 3:** Negative impacts of perceived stigma on the management of TB by patients.

Negative Impacts	No.	%
Feeling embarrassed/ashamed because of TB	166	61.0
Has the feelings of TB stigma prevented you from seeking and accessing TB services?	58	21.3
Experienced shame/embarrassment in hospitals or clinics that prevented continuing to seek and receive TB services?	60	22.1
Experienced shame or embarrassment from neighbors that prevented seeking and accessing TB services	52	19.1
Experienced shame/embarrassment at home that prevented seeking and obtaining TB services	38	14.0
Experienced a sense of shame/embarrassment at work that prevented seeking and obtaining TB services?	50	18.4
Experienced a sense of shame/embarrassment that prevented seeking timely care, receiving an accurate diagnosis, starting treatment, committing to treatment, or completing treatment	66	24.3
Denial of access to services for someone due to being infected with TB	70	25.7
Divorce of a woman due to being infected with TB	4	1.5
A family member with TB refused to disclose his condition	10	3.7
Wishing to see a change in TB services, laws, and policies to address the sense of shame and embarrassment related to TB	100	36.8

**Table 4:** Levels of perceived stigma among patients with TB according to their personal characteristics.

	Absent		Mild		Severe		*p*
Personal Characteristics	No.	%	No.	%	No.	%	Value
Age groups							
<25 years	20	32.8	5	8.2	36	59.0	
25–34 years	16	18.2	28	31.8	44	50.0	
35–44 years	18	23.4	23	29.9	36	46.8	0.011 †
45+	16	34.8	8	17.4	22	47.8	
Gender			64		138		
Male	46	24.0	48	25.0	98	51.0	
Female	24	30.0	16	20.0	40	50.0	0.493
Residence							
Urban	14	11.3	26	21.0	84	67.7	
Rural	56	37.8	38	25.7	54	36.5	<0.001 †
Marital status							
Single	42	29.2	28	19.4	74	51.4	
Married	26	23.6	32	29.1	52	47.3	
Divorced	0	0.0	2	25.0	6	75.0	0.327
Widow	2	20.0	2	20.0	6	60.0	
Occupation							
Unemployed	24	17.4	36	26.1	78	58.5	
Student	12	50.0	2	8.3	10	41.7	
Governmental job	14	25.9	12	22.2	28	51.9	0.009 †
Private job	18	37.5	10	20.8	20	41.7	
Retired	2	25.0	4	50.0	2	25.0	
Family monthly income							
<SAR 5000	50	29.1	38	22.1	84	48.8	
SAR 5000–10,000	14	24.1	14	24.1	30	51.7	0.402
>SAR 10,000	6	14.3	12	28.6	24	57.1	
Type of TB							
Pulmonary	50	23.4	44	20.6	120	56.1	
Extrapulmonary	20	34.5	20	34.5	18	31.0	0.003 †

† Statistically significant (*p* < 0.05).

**Table 5:** Aspects of perceived TB-related stigma among family members of patients with TB.

	Strongly Disagree		Disagree		Neutral		Agree		Strongly Agree	
Aspects of Perceived Stigma	No.	%	No.	%	No.	%	No.	%	No.	%
My family asks me to keep tuberculosis a secret	24	18.2	58	43.9	8	6.1	34	25.8	8	6.1
I’m ashamed that a member of my family has tuberculosis	32	24.2	60	45.5	14	10.6	16	12.1	10	7.6
I hide the fact that a member of my family has tuberculosis from the community	22	16.7	66	50.0	16	12.1	22	16.7	6	4.5
A family member hides his TB diagnosis from the community	28	21.2	58	43.9	22	16.7	18	13.6	6	4.5
I’m afraid someone will see me at the healthcare clinic where my relative is being treated	32	24.2	48	36.4	18	13.6	26	19.7	8	6.1
I’m afraid someone will see me at the healthcare clinic where my relative is being treated	34	25.8	62	47.0	6	4.5	22	16.7	8	6.1
Replace tuberculosis with another word in my conversations with a family member	26	19.7	60	45.5	14	10.6	26	19.7	6	4.5
Replace the word tuberculosis with another word in my conversations with my friends	26	19.7	58	43.9	12	9.1	30	22.7	6	4.5
I have noticed changes in a family member since he was diagnosed with tuberculosis	22	16.7	52	39.4	18	13.6	34	25.8	6	4.5
I’m worried about this disease	24	18.2	44	33.3	16	12.1	36	27.3	12	9.1

**Table 6:** Levels of perceived stigma among family members of patients with TB according to their personal characteristics.

	Absent		Mild		Severe		*p*
Personal Characteristics	No.	%	No.	%	No.	%	Value
Age groups							
<25 years	16	47.1	6	17.6	12	35.3	
25–34 years	24	54.5	12	27.3	8	18.2	
35–44 years	19	55.9	9	26.5	6	17.6	0.523
45+	8	40.0	6	30.0	6	30.0	
Gender							
Male	38	48.7	24	30.8	16	20.5	
Female	29	53.7	9	16.7	16	29.6	0.150
Residence							
Urban	14	29.2	14	29.2	20	41.7	
Rural	53	63.1	19	22.6	12	14.3	<0.001 †
Marital status							
Single	36	52.9	10	14.7	22	32.4	
Married	29	50.0	19	32.8	10	17.2	
Divorced	0	0.0	2	100.0	0	0.0	0.018 †
Widow	2	50.0	2	50.0	0	0.0	
Occupation							
Unemployed	33	47.1	17	24.3	20	28.6	
Student	6	50.0	2	16.7	4	33.3	
Governmental job	12	60.0	6	30.0	2	10.0	0.662
Private job	14	50.0	8	28.6	6	21.4	
Retired	2	100.0	0	0.0	0	0.0	
Family monthly income							
<SAR 5000	44	59.5	12	16.2	18	24.3	
SAR 5000–10,000	16	40.0	16	40.0	8	20.0	0.053
>SAR 10,000	7	38.9	5	27.8	6	33.3	
Type of TB							
Pulmonary	47	48.0	29	29.6	22	22.4	
Extrapulmonary	20	58.8	4	11.8	10	29.4	0.116

† Statistically significant (*p* < 0.05).

**Table 7:** Levels of perceived TB-related stigma according to study groups.

	Absent		Mild		Severe		*p*
Study Group	No.	%	No.	%	No.	%	Value
Patients with TB	70	25.7	64	23.5	138	50.7	
Family members	67	50.8	33	25.0	32	24.2	<0.001 †

† Statistically significant (*p* < 0.05).

## Data Availability

The raw data supporting the findings and conclusions of this study will be made available by the corresponding author upon reasonable request.
